# Morphometric Analysis of the Filum Terminale and Conus Medullaris: A Cadaveric Study

**DOI:** 10.7759/cureus.105233

**Published:** 2026-03-14

**Authors:** Priyanka N Sharma, Meghana H Joshi, Hetal Vaishnani, Priyanka Gohil, Kinjal V Jethva, Manoj M Kulkarni, Achleshwar R Gandotra

**Affiliations:** 1 Anatomy, Smt. B. K. Shah Medical Institute and Research Centre, Sumandeep Vidyapeeth Deemed to Be University, Vadodara, IND

**Keywords:** conus medullaris, extradural, filum terminale, intradural, spinal cord, tethered cord syndrome, vertebral column

## Abstract

Introduction

The filum terminale (FT) is a fibrous continuation of the spinal cord that anchors the conus medullaris to the coccyx. Variations in the termination level of the conus medullaris and morphometry of the FT are clinically relevant in the evaluation of tethered cord syndrome. However, comprehensive morphometric correlations between total spinal cord length (TLSC), vertebral column length, and FT dimensions remain limited in the literature.

Methods

Thirty formalin-fixed adult cadavers (15 males and 15 females) were dissected from C1 to Co1. Measurements included TLSC, length up to the conus medullaris (LSCCM), total filum terminale length (FT-L), internum (FTI-L) and externum (FTE-L) lengths, segmental diameters, vertebral column length (VCL), and cadaver height (CH). Vertebral termination levels of the conus medullaris and FT-dural sac (DS) fusion were documented. Statistical analysis was performed using independent t-tests, Pearson correlation, and multiple linear regression.

Results

The conus medullaris most frequently terminated at the lower third of L1. The FT fused with the DS predominantly at the upper third of S2. The mean FT length was 221.83 ± 24.90 mm, with all segmental diameters remaining below the pathological 2 mm threshold. Significant positive correlations were observed between the VCL and TLSC (p < 0.001), TLSC and LSCCM (p < 0.001), and FT-L and proximal intradural diameter (p < 0.001). CH was strongly correlated with TLSC length and selected FT parameters. Significant sex differences were identified in the TLSC, LSCCM, selected diameter measurements, CH, and VCL.

Conclusion

This study establishes normative cadaveric morphometric data for the conus medullaris and FT. The novel integration of TLSC, VCL, CH, and detailed FT morphometry demonstrates proportional anatomical scaling. These findings enhance anatomical understanding and provide clinically relevant reference values for distinguishing physiological variations from pathological tethering in tethered cord syndrome.

## Introduction

The development of the filum terminale (FT) is a result of dedifferentiation occurring in the caudal region of the embryonic and fetal spinal cord (SC) [1]. Initially, the SC fills the entire space within the dura mater. As the skeleton matures, the vertebral bodies undergo ossification, and the spine lengthens. During embryonic growth, the vertebral column extends, but the SC grows at a slower pace. The SC occupies the upper two-thirds of the vertebral canal and ends caudally as the conus medullaris (CM) [2]. In the later stages of fetal development, the CM is located between L3 and S5. In premature and full-term newborns, it is found between L1-L3 [3], and in children aged one to seven years, it is situated between T12 and L3. In adults, the SC typically ends at the middle third of the L1 vertebra. Following the termination of the CM, a fibrous band known as the FT connects the CM to the coccyx [3-7]. The length of the CM and FT is directly related to body length, although slight variations can occur among individuals of the same height. The entire FT is approximately 20 cm long and is composed of glial and ependymal cells [4,8-11]. Luschka was the first to describe the two distinct segments of the FT. Beginning at the tip of the CM, the filum terminale internum (FTI) extends within the caudal extension of the dural sac (DS). It eventually pierces or merges with the dura mater [4,6,11,12]. The filum terminale externum (FTE), also known as the coccygeal ligament, continues from the distal FT and measures approximately 5 cm in length, where it fuses with the dura mater. It then connects the distal dural cul-de-sac to the periosteum of the coccyx [3-5,7,11-13]. The sacral canal also contains the cauda equina and the spinal meninges. At the level opposite the middle of the sacrum (S2), the lower sacral spinal roots and FT fuse with the dura mater [6,8,14]. The FT, along with its meningeal coverings, emerges below the sacral hiatus and descends across the dorsal surface of S5 and the sacrococcygeal joint to reach the coccyx, with the first coccygeal nerve running alongside the filum [4].

The anatomical configuration of the spinal structure, particularly the termination level of the conus medullaris (CMT), is of considerable clinical significance to anesthesiologists and spine surgeons. A comprehensive understanding of the CMT level is crucial for enhancing the safety of high-level lumbar anesthesia, thereby mitigating the risk of SC injury [15]. Approximately 15% of SC injuries occur in the thoracolumbar region, resulting in conditions such as CM or cauda equina (CE) syndromes. These conditions are characterized by symptoms, including low back pain, weakness in the lower limbs, anesthesia in the perineum or saddle area, and bowel and/or bladder dysfunction [16]. Tethered cord syndrome (TCS) is a condition characterized by pathological traction on the SC [17,18]. TCS was first described by Garceau in 1953 as “filum terminale syndrome” [17] and later termed TCS by Hoffman and colleagues in 1976 [18]. The pathophysiology is believed to involve reduced elasticity or abnormal thickening of the FT, leading to impaired SC mobility and progressive neurological symptoms. Even a radiologically normal-appearing FT may exert traction if its intrinsic structure is altered [9]. TCS has been associated with a spectrum of neurological deficits, including motor weakness, gait abnormalities, and bladder dysfunction [4,19].

The primary objective of this study was to evaluate the morphometric characteristics of the FT and CM in adult cadavers. The specific objectives were to determine the vertebral level of termination of the CM, measure the filum terminale length (FT-L) and its intradural and extradural components, assess segmental diameter variations along the FT, and identify the level of fusion of the FT with the DS. In addition, the study aimed to analyse sex-based differences and evaluate correlations between FT measurements, vertebral column length (VCL), total spinal cord length (TLSC), and cadaver height (CH). The scope was to establish comprehensive normative data and clarify anatomical relationships relevant to tethered cord syndrome. To our knowledge, the relationship between VCL, CH, and FT has not been extensively documented in the current literature.

## Materials and methods

Thirty formalin-fixed adult embalmed cadavers (15 males and 15 females) with a mean age at death of 77 years underwent dissection of the entire length of the vertebral column to examine the entire length of the SC and FT. The present study was conducted at the Anatomy Department of Smt. B. K. Shah Medical Institute and Research Centre, Vadodara, Gujarat, India. Ethical approval for the present study was obtained from the Institutional Ethics Committee (Sumandeep Vidyapeeth Institutional Ethics Committee, Reference Letter No.- SVIEC/OW/MEDI/PHD/18005).

**Figure 1:**
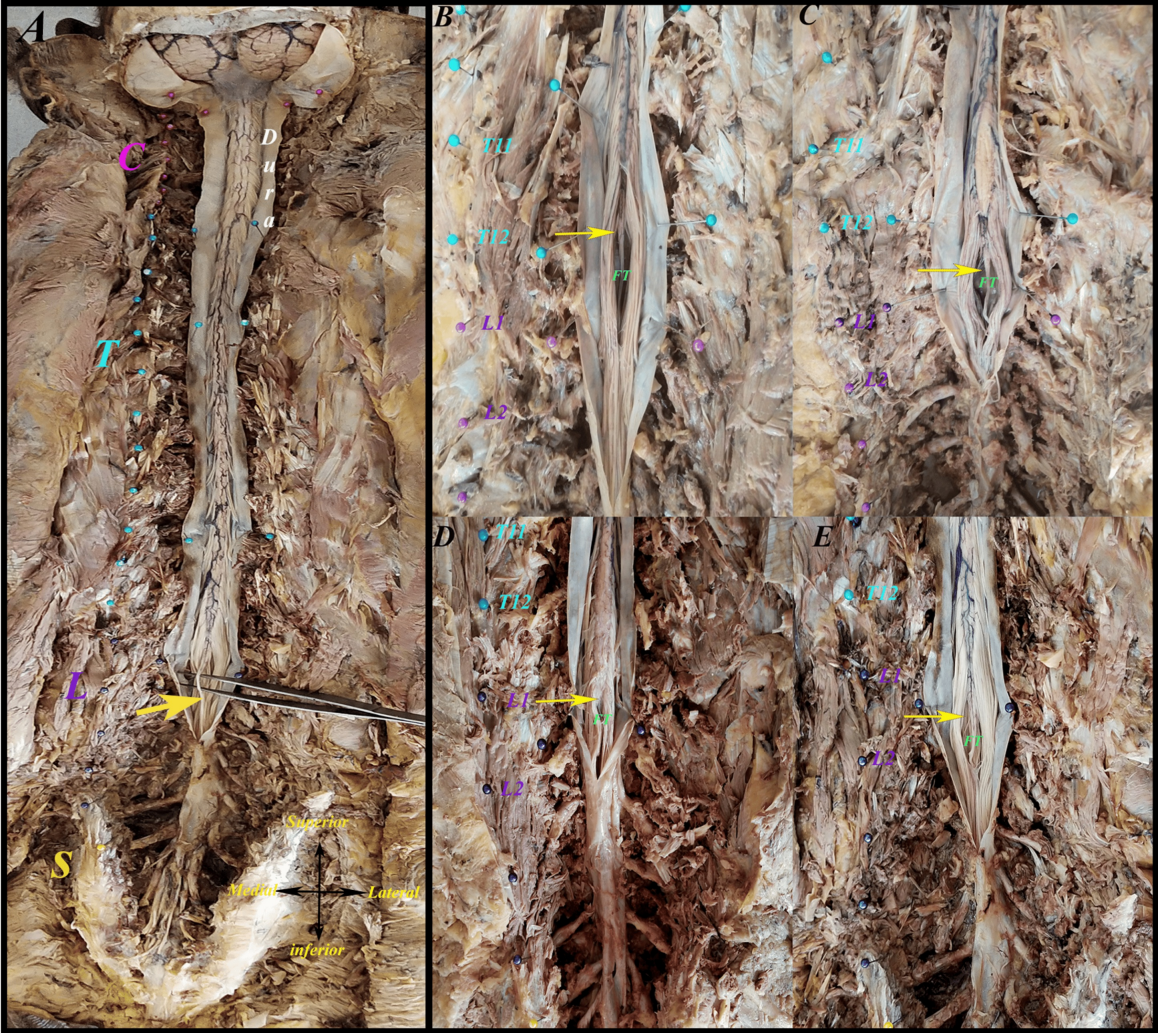
(A) Illustration of the spinal cord and FT (yellow arrow) in a cadaver. (B-E) Figures showing different vertebral level termination of conus medullaris (yellow arrows). (B) Middle third T12, (C) T12-L1 disc, (D) Middle third L1, (E) Lower third L1 C: cervical, T: thoracic, L: lumbar, S: sacral, FT: filum terminale

**Figure 2:**
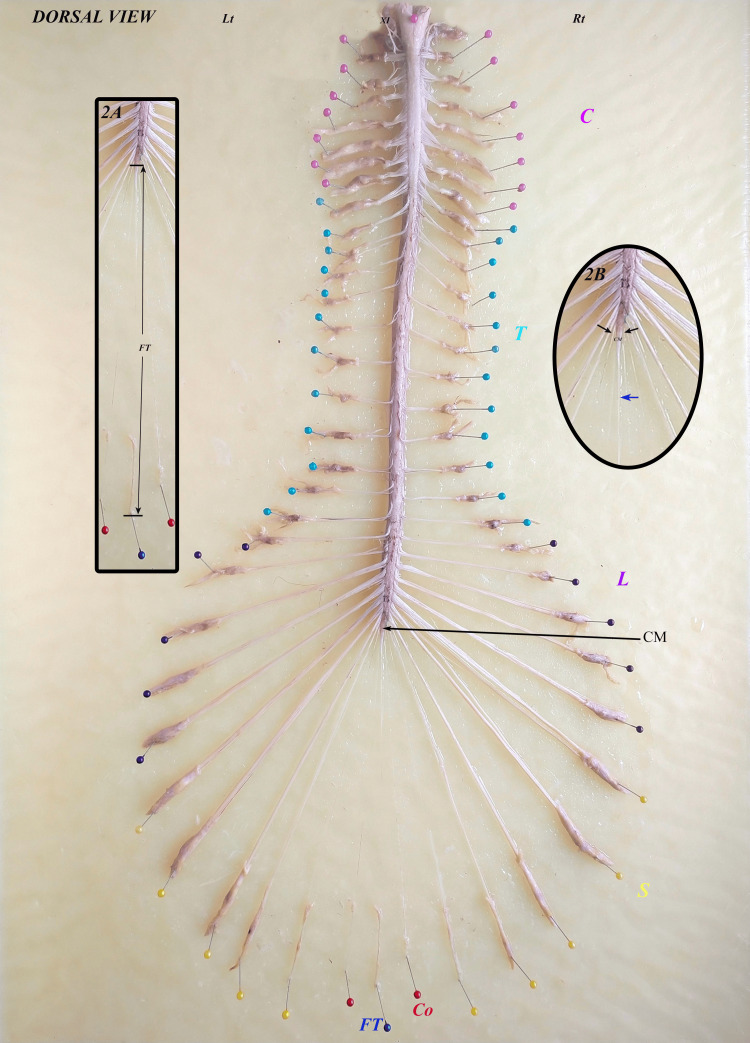
Spinal cord, conus medullaris (black arrow), and FT after being placed on a wax board. (A) FT, (B) Magnifying view of conus medullaris (last pair of coccygeal with black arrow, FT with blue arrow) Lt: left, Rt: right, CM: conus medullaris, C: cervical, T: thoracic, L: lumbar, S: sacral, Co: coccygeal, FT: filum terminale

**Figure 3:**
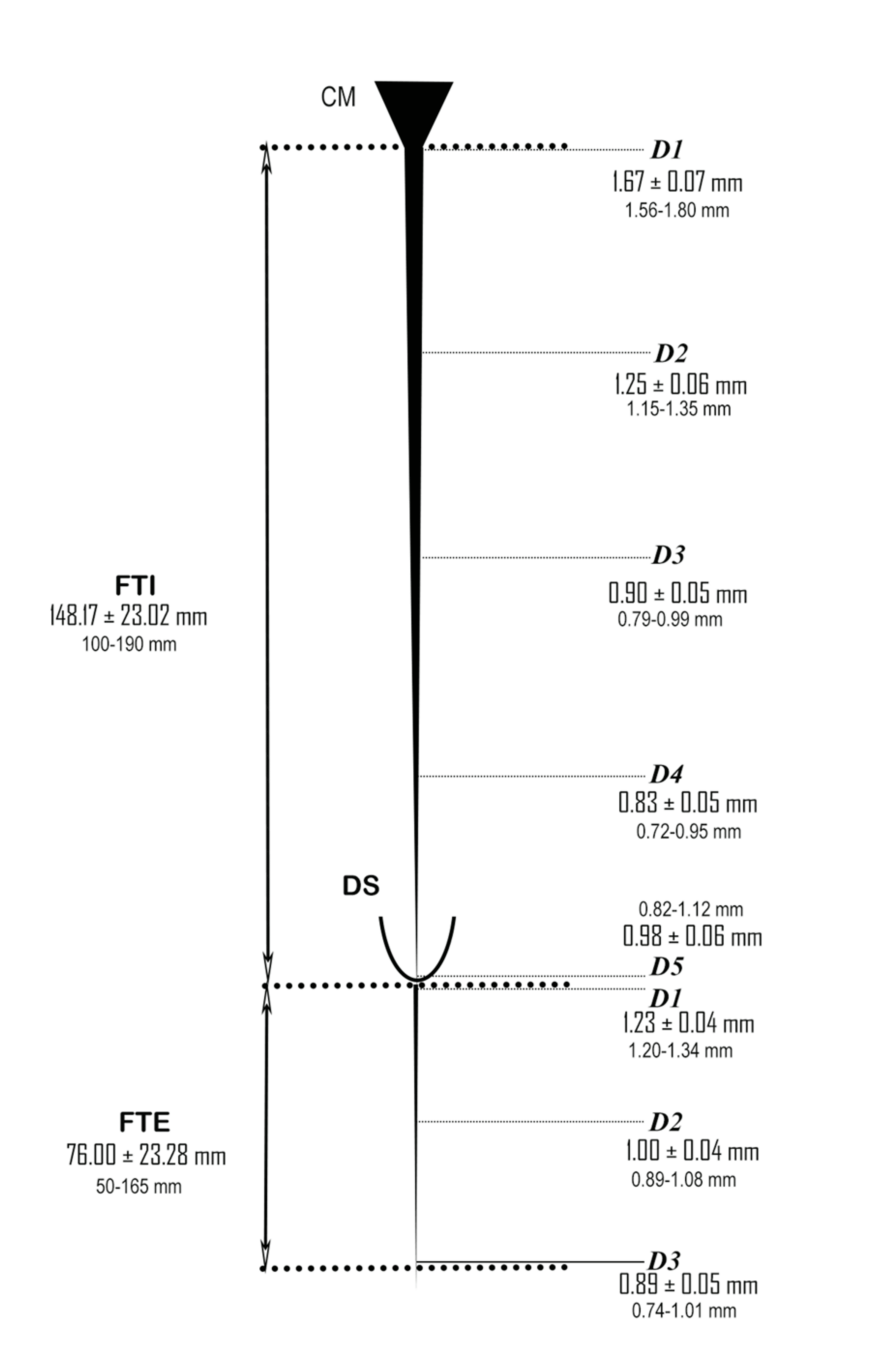
Schematic diagram representing measurements of filum terminale with mean value of each variable. FTI: filum terminale internum, FTE: filum terminale externum length, FTI-D1 to D5: segmental diameters of filum terminale internum; FTE-D1 to D3: segmental diameters of filum terminale externum, CM: conus medullaris, DS: dural sac

The vertebral column was dorsally dissected in 30 specimens without any previous history of spine surgery, infection, trauma, tumor, deformity, metastasis, or severe osteoporosis; spondylolisthesis was excluded from this study. A midline skin incision was made over the cervical spine to the coccyx with the cadavers in the prone position. The vertebral spines were exposed, and vertebrectomies were performed from C1 to Co1 vertebral levels. A longitudinal incision was made in the mid-dorsal region of the DS along the exposed area. The CM was traced downward to identify the start of the FT after the last pair of coccygeal nerve roots exited the conus. The vertebrae were divided into three portions (upper, middle, and lower thirds), and the intervertebral disc space was also considered for this parameter, as explained by Hansasuta et al. [7]. The termination level of the CM relative to the vertebrae was documented (Figure 1). The initial point of the FT was identified as the junction with the CM, as described by Reimann and Anson [20]. The FT was identified and followed caudally until it pierced or fused with the dura. The FTI was observed from the initial point of the FT to its fusion with the DS. The gross appearance of the filum and its fusion site (midline, left, or right) was recorded. The DS was also traced to its termination. No adhesions were observed between the FT and surrounding structures. The levels of dural fusion of the filum and the termination of the DS were recorded. The fusion level of the FT and the termination of the DS were determined in relation to the sacral segments. The termination of the filum was observed at the coccygeal segments (Figure 1). The FTE was identified starting at the apex of the DS and traced distally to its attachment at the dorsal coccyx (Figure 2). Subsequently, the FT was carefully removed en bloc with a collar of dura mater to preserve the FT-DS junction. Caudally, a fragment of coccygeal periosteum was detached to ensure the integrity of the FTE and mounted on a wax block for further analysis (Figure 2). All observations were recorded in a standardized format, and the length and diameters of the FT, FTI, and FTE were measured using a digital Vernier caliper (Figure 3).

Data collection and analysis were performed using IBM SPSS Statistics for Windows, Version 23 (Released 2016; IBM Corp., Armonk, New York, United States). Continuous variables were expressed as the mean ± standard deviation. Normality was assessed using the Shapiro-Wilk test. As most variables were normally distributed, parametric tests were applied. An independent samples t-test was used for sex-wise comparisons. Pearson’s correlation coefficient was used to assess linear relationships between morphometric parameters. Multiple linear regression analysis was performed to identify predictors of proximal intradural diameter (FTI-D1). The chi-square test was used to evaluate associations between categorical variables. A p-value < 0.05 was considered statistically significant.

## Results

Thirty adult cadavers were included in this study. The morphometric measurements of SC and FT are summarized in Table 1.

**Table 1:** Measurements of the spinal cord and filum terminale in cadavers. TLSC: total length of spinal cord, LSCCM: length of spinal cord up to conus medullaris, FT-L: filum terminale length, FTI-L: filum terminale internum length, FTE-L: filum terminale externum length, CH: cadaver height, VCL: vertebral column length, FTI-D1 to FTI-D5: segmental diameters of filum terminale internum; FTE-D1 to FTE-D3: segmental diameters of filum terminale externum, CMT: conus medullaris termination, LL1: lower third L1, M12: middle third 12, DS: dural sac

Parameters	Mean ± SD	Minimum	Maximum
TLSC (mm)	613.67 ± 47.21	520.00	710.00
LSCCM (mm)	391.50 ± 33.38	310.00	440.00
FT-L (mm)	221.83 ± 24.90	180.00	290.00
FTI-L (mm)	148.17 ± 23.02	100.00	190.00
FTE-L (mm)	76.00 ± 23.28	50.00	165.00
FTI-D1 (Proximal) (mm)	1.67 ± 0.07	1.56	1.80
FTI-D2 (mm)	1.25 ± 0.06	1.15	1.35
FTI-D3 (mm)	0.90 ± 0.05	0.79	0.99
FTI-D4 (mm)	0.83 ± 0.05	0.72	0.95
FTI-D5 (Distal intradural) (mm)	0.98 ± 0.06	0.82	1.12
FTE-D1 (Proximal extradural) (mm)	1.23 ± 0.04	1.20	1.34
FTE-D2 (mm)	1.00 ± 0.04	0.89	1.08
FTE-D3 (Distal extradural) (mm)	0.89 ± 0.05	0.74	1.01
CH (cm)	161.93 ± 6.84	148.00	172.00
VCL (cm)	65.00 ± 4.28	58.00	71.00
CMT	LL1	M12	L2-L3 disc
Fusion of FT to DS	Upper third S2	L5-S1 disc	Upper third S3

**Figure 4:**
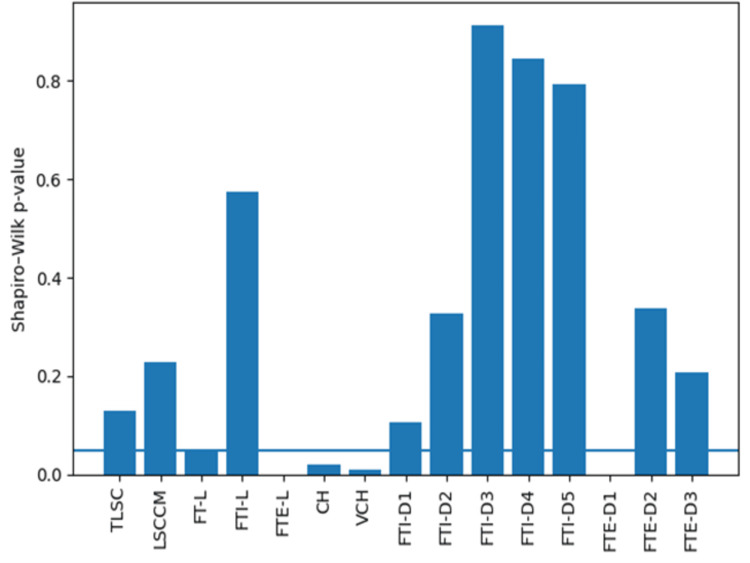
Shapiro-Wilk test demonstrating normal distribution of morphometric variables. TLSC: total length of spinal cord, LSCCM: length of spinal cord up to conus medullaris, FT-L: filum terminale length, FTI-L: filum terminale internum length, FTE-L: filum terminale externum length, CH: cadaver height, VCL: vertebral column length, FTI-D1 to FTI-D5: segmental diameters of filum terminale internum; FTE-D1 to FTE-D3: segmental diameters of filum terminale externum

Normality of continuous variables was assessed using the Shapiro-Wilk test (Figure 4). The majority of the morphometric parameters demonstrated p-values greater than 0.05, indicating no significant deviation from the normal distribution. Although a small number of variables showed p-values near or below the threshold, the overall distribution patterns were consistent with approximate normality. Therefore, parametric statistical tests were considered appropriate for subsequent comparative and correlation analyses.

**Figure 5:**
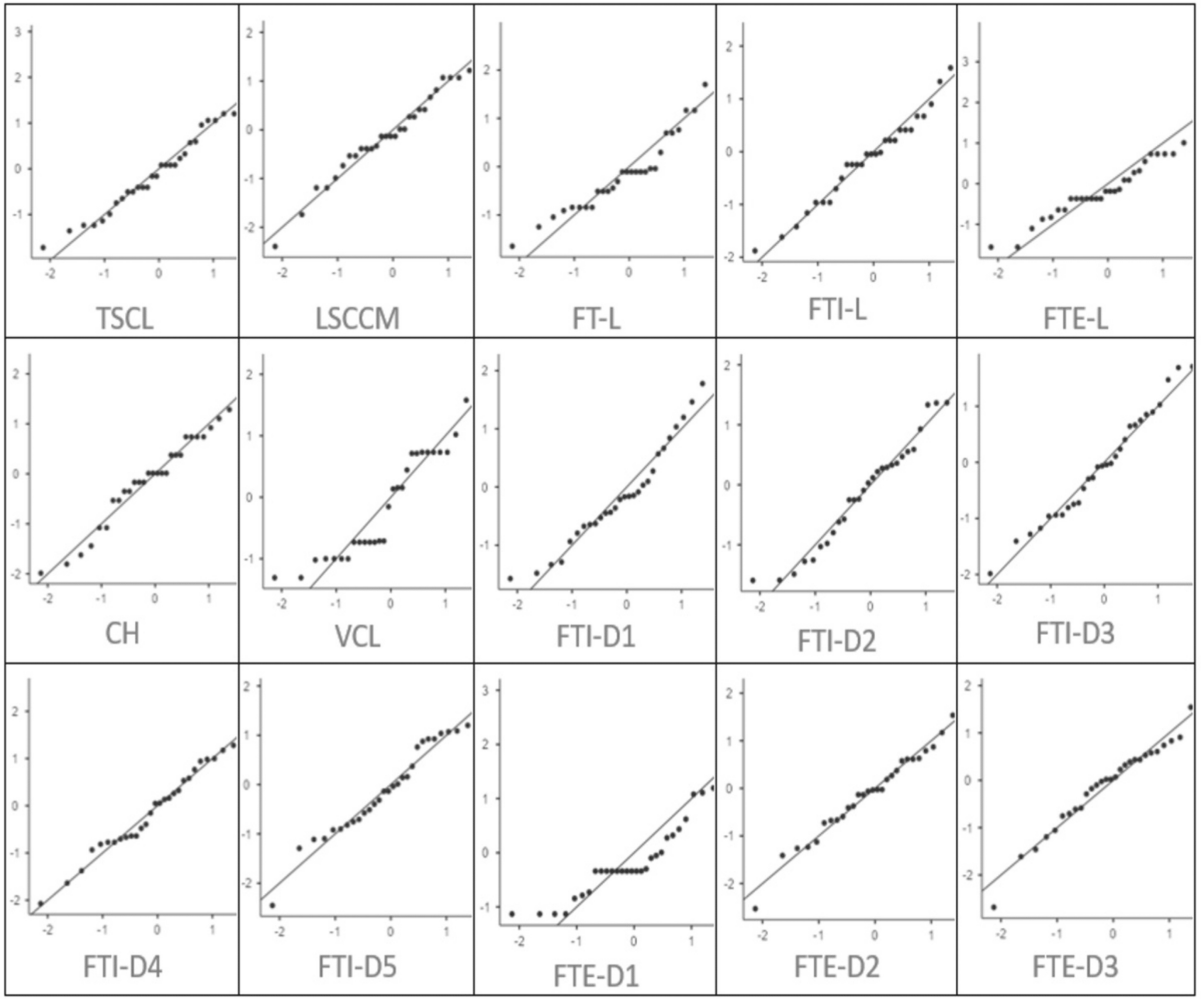
Q-Q plots demonstrating normal distribution of morphometric variables. TLSC: total length of spinal cord, LSCCM: length of spinal cord up to conus medullaris, FT-L: filum terminale length, FTI-L: filum terminale internum length, FTE-L: filum terminale externum length, CH: cadaver height, VCL: vertebral column length, FTI-D1 to FTI-D5: segmental diameters of filum terminale internum; FTE-D1 to FTE-D3: segmental diameters of filum terminale externum

A graphical assessment using Q-Q plots further supported these findings (Figure 5). Most variables demonstrated data points closely aligned along the reference diagonal line, with only minor deviations at the extreme quantiles. No substantial skewness or kurtosis was observed, reinforcing the suitability of the parametric methods.

The mean TLSC was 613.67 ± 47.21 mm (range: 520-710 mm), whereas the mean LSCCM was 391.50 ± 33.38 mm (range: 310-440 mm). The total length of the FT (FT-L) was 221.83 ± 24.90 mm (range: 180-290 mm), comprising an intradural portion (FTI-L) of 148.17 ± 23.02 mm (range: 100-190 mm) and an extradural portion (FTE-L) of 76.00 ± 23.28 mm (range: 50-165 mm). Segmental diameter analysis of the FTI revealed a tapering pattern. The proximal diameter (FTI-D1) was 1.67 ± 0.07 mm (range: 1.56-1.80 mm), decreasing to 1.25 ± 0.06 mm (range: 1.15-1.35 mm) at FTI-D2, 0.90 ± 0.05 mm (range: 0.79-0.99 mm) at FTI-D3, and 0.83 ± 0.05 mm (range: 0.72-0.95 mm) at FTI-D4, followed by a mild distal increase at FTI-D5 (0.98 ± 0.06 mm, range: 0.82-1.12 mm). The FTE showed a proximal diameter (FTE-D1) of 1.23 ± 0.04 mm (range: 1.20-1.34 mm), decreasing to 1.00 ± 0.04 mm (range: 0.89-1.08 mm) at FTE-D2 and 0.89 ± 0.05 mm (range: 0.74-1.01 mm) at FTE-D3. The mean CH was 161.93 ± 6.84 cm (range: 148-172 cm), and the VCL was 65.00 ± 4.28 cm (range: 58-71 cm). The CM most commonly terminated at the lower third of L1 (LL1) (Figure 1), with observed variation from the middle third T12 (MT12) to L2-L3 discs.

Fusion of the FT to the DS was predominantly observed at the upper third of the S2 in the midline, with variations extending from the L5-S1 disc level to the lower third of the S2 (Table 1).

**Figure 6:**
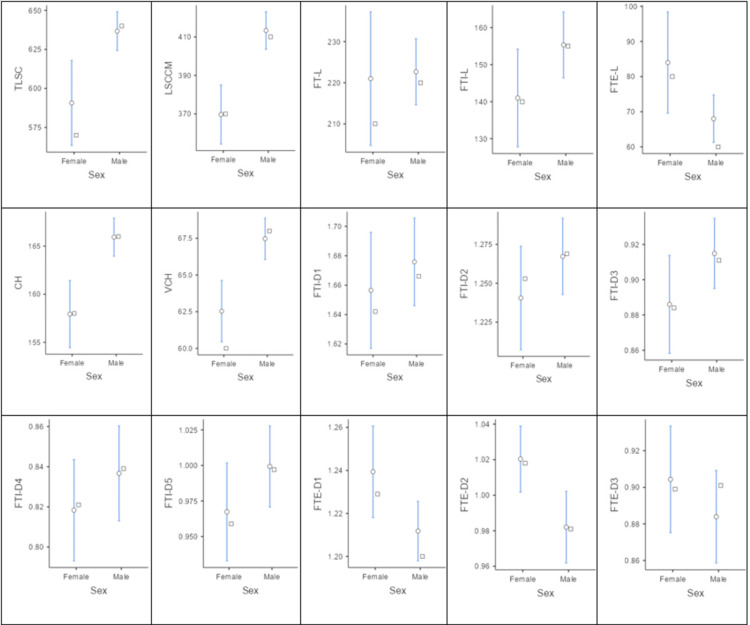
Sex-based comparison of spinal cord and filum terminale morphometric variables. TLSC: total length of spinal cord, LSCCM: length of spinal cord up to conus medullaris, FT-L: filum terminale length, FTI-L: filum terminale internum length, FTE-L: filum terminale externum length, CH: cadaver height, VCL: vertebral column length, FTI-D1 to FTI-D5: segmental diameters of filum terminale internum; FTE-D1 to FTE-D3: segmental diameters of filum terminale externum

Sex-wise analysis revealed that males had a significantly greater TLSC (636.14 ± 24.39 mm) than females (590.67 ± 53.75 mm; p = 0.003). Lumbosacral canal-conus medullaris length (LSCCM) was also significantly higher in males (413.33 ± 19.94 mm vs. 369.67 ± 30.26 mm; p < 0.001). CH and VCL were significantly greater in males (p < 0.001 for both). Among the diameter measurements, FTI-D3 was significantly higher in males (0.92 ± 0.04 mm) than in females (0.89 ± 0.05 mm; p = 0.04). Conversely, females demonstrated slightly greater extradural diameters at FTE-D1 (1.24 ± 0.04 mm vs. 1.21 ± 0.03 mm; p = 0.02) and FTE-D2 (1.02 ± 0.04 mm vs. 0.98 ± 0.04 mm; p = 0.01). No significant sex-based differences were observed in total FT length, intradural FT length, extradural FT length, or other diameter measurements (p > 0.05) (Figure 6 and Table 2).

**Table 2:** Measurements of the spinal cord and filum terminale in all cadavers and in male and female cadavers. TLSC: total length of spinal cord, LSCCM: length of spinal cord up to conus medullaris, FT-L: filum terminale length, FTI-L: filum terminale internum length, FTE-L: filum terminale externum length, CH: cadaver height, VCL: vertebral column length, FTI-D1 to FTI-D5: segmental diameters of filum terminale internum; FTE-D1 to FTE-D3: segmental diameters of filum terminale externum, CMT: conus medullaris termination, LL1: lower third L1, M12: middle third 12, DS: dural sac * Statistically significant difference between males and females (p < 0.05)

Variable	Male (Mean ± SD)	Female (Mean ± SD)	Overall (Mean ± SD)	P-value
TLSC (mm)	636.14 ± 24.39	590.67 ± 53.75	613.67 ± 47.21	0.003*
LSCCM (mm)	413.33 ± 19.94	369.67 ± 30.26	391.50 ± 33.38	<0.001*
FT-L (mm)	222.67 ± 15.90	221.00 ± 32.08	221.83 ± 24.90	0.84
FTI-L (mm)	155.33 ± 17.57	141.00 ± 26.06	148.17 ± 23.02	0.09
FTE-L (mm)	68.00 ± 13.33	84.00 ± 28.42	76.00 ± 23.28	0.06
FTI-D1 (Proximal) (mm)	1.68 ± 0.06	1.66 ± 0.08	1.67 ± 0.07	0.42
FTI-D2 (mm)	1.27 ± 0.05	1.24 ± 0.07	1.25 ± 0.06	0.18
FTI-D3 (mm)	0.92 ± 0.04	0.89 ± 0.05	0.90 ± 0.05	0.04*
FTI-D4 (mm)	0.84 ± 0.05	0.82 ± 0.05	0.83 ± 0.05	0.21
FTI-D5 (Distal intradural) (mm)	1.00 ± 0.06	0.97 ± 0.07	0.98 ± 0.06	0.19
FTE-D1 (Proximal extradural) (mm)	1.21 ± 0.03	1.24 ± 0.04	1.23 ± 0.04	0.02*
FTE-D2 (mm)	0.98 ± 0.04	1.02 ± 0.04	1.00 ± 0.04	0.01*
FTE-D3 (Distal extradural) (mm)	0.88 ± 0.05	0.90 ± 0.06	0.89 ± 0.05	0.32
CH (cm)	165.93 ± 4.04	157.93 ± 6.89	161.93 ± 6.84	<0.001*
VCL (cm)	67.46 ± 2.77	62.53 ± 4.14	65.00 ± 4.28	<0.001*
CMT	LL1	LL1	LL1	-
Fusion of FT to DS	Upper third S2	Upper third S2	Upper third S2	-

Pearson’s correlation analysis demonstrated a strong positive correlation between the total FT length and intradural length (r = 0.747, p < 0.001). The TLSC was strongly correlated with the length up to the CM (r = 0.866, p < 0.001). Significantly strong intersegmental correlations were observed among intradural diameters, particularly between FTI-D1 and FTI-D2 (r = 0.780, p < 0.001) and between FTI-D3 and FTI-D4 (r = 0.874, p < 0.001). Extradural diameters also showed significant positive correlations, especially between FTE-D1 and FTE-D2 (r = 0.692, p < 0.001). The total FT-L was strongly correlated with the proximal intradural diameter (FTI-D1) (r = 0.724, p < 0.001). CH and VCL demonstrated strong positive correlations with TLSC (r = 0.827 and r = 0.846, respectively; p < 0.001) (Table 3).

**Table 3:** Pearson correlation matrix of spinal cord and filum terminale morphometric variables. TLSC: Total length of spinal cord, LSCCM: length of spinal cord up to the conus medullaris, FT-L: filum terminale length, FTI-L: filum terminale internum length, FTE-L: filum terminale externum length, CH: cadaver height, VCL: vertebral column length, FTI-D1 to FTI-D5: segmental diameters of filum terminale internum, FTE-D1 to FTE-D3: segmental diameters of filum terminale externum, CMT: conus medullaris termination, LL1: lower third L1, M12: middle third 12, DS: dural sac Values represent Pearson’s correlation coefficients (r). Statistical significance: * p < 0.05, ** p < 0.005, *** p < 0.001. Based on the model developed by Buloz-Osorio et al. [10] and De Vloo et al. [12].

	TLSC	LSCCM	FT-L	FTI-L	FTE-L	CH	VCL	FTI-D1	FTI-D2	FTI-D3	FTI-D4	FTI-D5	FTE-D1	FTE-D2	FTE-D3
TLSC	1.000***	0.866***	0.743***	0.684***	0.330	0.827***	0.846***	0.613***	0.571***	0.481**	0.353	0.362*	0.169	0.036	0.340
LSCCM	0.866***	1.000***	0.310	0.404*	0.104	0.712***	0.791***	0.332	0.403*	0.429*	0.310	0.301	-0.034	-0.171	0.147
FT-L	0.743***	0.310	1.000***	0.747***	0.495**	0.628***	0.546**	0.724***	0.542**	0.324	0.244	0.265	0.375*	0.309	0.460*
FTI-L	0.684***	0.404*	0.747***	1.000***	0.205	0.539**	0.506**	0.589***	0.489**	0.342	0.203	0.270	0.105	0.179	0.375*
FTE-L	0.330	0.104	0.495**	0.205	1.000***	0.207	0.087	0.459*	0.195	0.102	0.216	0.088	0.824***	0.504**	0.510**
CH	0.827***	0.712***	0.628***	0.539**	0.207	1.000***	0.871***	0.588***	0.636***	0.492**	0.410*	0.466**	0.085	-0.055	0.248
VCL	0.846***	0.791***	0.546**	0.506**	0.087	0.871***	1.000***	0.368*	0.497**	0.448*	0.367*	0.470**	-0.062	-0.147	0.150
FTI-D1	0.613***	0.332	0.724***	0.589***	0.459*	0.588***	0.368*	1.000***	0.780***	0.623***	0.506**	0.467**	0.417*	0.337	0.423*
FTI-D2	0.571***	0.403*	0.542**	0.489**	0.195	0.636***	0.497**	0.780***	1.000***	0.765***	0.633***	0.666***	0.256	0.131	0.267
FTI-D3	0.481**	0.429*	0.324	0.342	0.102	0.492**	0.448*	0.623***	0.765***	1.000***	0.874***	0.834***	-0.034	-0.013	0.117
FTI-D4	0.353	0.310	0.244	0.203	0.216	0.410*	0.367*	0.506**	0.633***	0.874***	1.000***	0.926***	0.126	0.047	0.164
FTI-D5	0.362*	0.301	0.265	0.270	0.088	0.466**	0.470**	0.467**	0.666***	0.834***	0.926***	1.000***	0.052	-0.028	0.076
FTE-D1	0.169	-0.034	0.375*	0.105	0.824***	0.085	-0.062	0.417*	0.256	-0.034	0.126	0.052	1.000***	0.692***	0.615***
FTE-D2	0.036	-0.171	0.309	0.179	0.504**	-0.055	-0.147	0.337	0.131	-0.013	0.047	-0.028	0.692***	1.000***	0.784***
FTE-D3	0.340	0.147	0.460*	0.375*	0.510**	0.248	0.150	0.423*	0.267	0.117	0.164	0.076	0.615***	0.784***	1.000***

Multiple linear regression analysis identified total FT-L as a significant predictor of proximal intradural diameter (FTI-D1) (p < 0.05). CH contributed to the model, whereas sex was not an independent predictor after adjustment. The regression model demonstrated a significant overall fit. Chi-square analysis revealed no significant association between sex and vertebral level of CMT (p = 0.14), indicating similar termination patterns between males and females.

Overall, the findings demonstrated coordinated morphometric scaling of the FT and SC, with limited but significant sex-related differences in the selected parameters. Clinically, these morphometric relationships may assist in distinguishing physiological variations from pathological thickening of the FT in tethered cord syndrome.

## Discussion

TCS represents a pathological state of SC traction resulting from restricted mobility of the distal SC, most commonly due to abnormalities of the FT [17,18,21]. The underlying mechanism is believed to involve decreased elasticity or abnormal thickening of the FT, resulting in chronic tension on the CM [9,12,21]. Although traditionally associated with a low-lying CM, increasing evidence suggests that tethering may occur even when the conus terminates at a normal vertebral level, implicating the FT as the primary pathological structure. An FT diameter greater than 2 mm is widely regarded as a radiological indicator of abnormal thickening [21]. However, De Vloo et al. [12] proposed that even a radiologically normal FT may generate pathological traction if its intrinsic architecture is altered by increased fibrous tissue content, resulting in reduced elasticity. Therefore, establishing normative morphometric values is critical for distinguishing physiological variations from pathological thickening.

The morphometric findings of the present study are largely consistent with those of earlier cadaveric and MRI-based investigations, although certain quantitative variations were observed. In the current series (n = 30), the CM most commonly terminated at the lower third of L1, with a range from the middle third of T12 to the L2-L3 disc level. This closely parallels the classical anatomical descriptions by Thomson [22] and McCotter [23], both of whom identified the lower third of L1 as the predominant termination level. Similar observations were reported by Reimann and Anson [20], Nasr [24], and De Vloo et al. [12]. Needles [25] and Gaddam et al. [3] also reported most frequently at the upper third of L2. Large MRI-based studies, including Soleiman et al. [26], Liu et al. [15], Macdonald et al. [27], and Jung JY et al. [28], documented a termination range extending from T12 to L3, further confirming that the present findings fall well within established anatomical limits. Fusion of the FT to the DS in the present study occurred most frequently at the upper third of S2, with variations from the L5-S1 disc to the upper third of S3. This is in close agreement with Nasr [14] and Nasr et al. [8], both of whom reported predominant fusion at S2. Buloz-Osorio et al. [10] similarly observed S2 as the most frequent fusion level, whereas Pokanan et al. [6] described slightly broader sacral variation extending toward S3 (Table 4). Thus, the sacral fixation pattern observed in the present study aligns with previously reported cadaveric data. Furthermore, the predominant fusion of the FT to the DS at the upper third of S2 provides important anatomical reference data for sacral surgical approaches and the interpretation of imaging findings.

**Table 4:** Terminale levels of conus medullaris and fusion of FT to dural sac comparison with other studies. FT: filum terminale, SI: stillborn infants, E: embalmed, F: fresh, DS: dural sac, MRI: magnetic resonance imaging

Authors	Study type	Sample size	Conus medullaris termination	Range of conus medullaris termination	Fusion of FT to DS	Range fusion of FT to DS
Thomson A (1894) [22]	Unspecified	198	Lower third L1	Middle third T12 - Upper third L3	-	-
McCotter RE (1916) [23]	Unspecified	234	Lower third L1	Lower third T12 - Upper third L3	-	-
Needles JH (1935) [25]	Cadavers	240	Upper third L2	Middle third T12 - Lower third L3	-	-
Reimann and Anson et al. (1944) [20]	Cadavers	129	Lower third L1	Lower third T12 - Middle third L3	-	-
Hansasuta A et al. (1999) [7]	Cadavers	27	-	-	-	-
Macdonald A et al. (1999) [27]	MRI	136	Middle third L1	Middle third T11 - Middle third L3	-	-
Pinto FC et al. (2002) [5]	Cadavers (Fresh)	41	Middle third L1	Lower third T11 - L2-L3 disc	-	-
Soleiman J et al. (2005) [26]	SMRI	635	Middle third L1	Lower third T11 - Upper third L3	-	-
Nasr AY (2007) [14]	Cadavers	10	L1-2 disc	Lower third T12 - Middle third L2	Upper third S2	L5-S1 disc - Lower third S2
	MRI	66	L1-2 disc	T12 - L3-4 disc	-	-
Gaddam SS et al. (2012) [3]	Cadavers (SI)	13	Upper third L2	Lower third L1 – Upper third L2	-	-
Nasr AY (2016) [24]	MRI	200	Lower third L1	Lower third T12 - L2-3 disc	-	-
De Vloo P et al. (2015) [12]	Cadavers (Fresh and Embalmed)	20	Lower third L1	Middle third T12 - Upper third L3	-	-
Jung JY et al. (2016) [28]	MRI	350	Lower third L1	Upper third T12 - Upper third L4	-	-
Nasr AY (2016) [24]	Cadavers	60	Lower third L1	Lower third T12 - L2-3 disc	-	-
	MRI	200	L1-L2 disc	Lower third T12 - L2-3 disc	-	-
Nasr AY et al. (2018) [8]	Cadavers	25	Lower third L1	-	Upper third S2	-
	MRI	100	L1-2 disc	-	Upper third S2	-
Liu A et al. (2017) [15]	MRI	585	Lower third L1	Lower third T12 - Upper third L3	-	-
Pokanan S et al. (2019) [6]	Cadavers (E)	83	-	-	S1/S2 disc	Upper third L5 - Upper third S3
Picart T et al. (2019) [1]	Cadavers (F)	10	Lower third of L1 and L1-L2 disc	Lower third of T12 - L2-L3 disc	-	-
Buloz-Osorio EB et al. (2025) [10]	Cadavers (E)	38	L1-2 disc	-	Upper third S2	-
Present Study	Cadavers (E)	30	Lower third L1	Middle third T12 - L2-l3 disc	Upper third S2	L5-S1 disc - Upper third S3

The mean total FT length in the present study was 221.83 ± 24.90 mm. This value is intermediate when compared with those of Nasr (247.3 ± 35.2 mm) [14] and Buloz-Osorio et al. (254.32 ± 26.46 mm) [10], but slightly greater than that of Nasr et al. (202.9 ± 20.9 mm) [8] in embalmed specimens. Gaddam et al. [3] observed 29 mm FT-L in stillborn infants. These differences may reflect methodological variability, embalming effects, or geographically specific anatomical differences. The intradural filum length (148.17 ± 23.02 mm) in the present study is comparable to that reported by Pinto et al. (156.44 mm) [5], Fontes et al. (155.4 mm) [29], Nasr et al. (162.3 ± 12.8 mm) [8], and De Vloo et al. (160.01 mm) [12], indicating strong consistency across independent investigations. Similarly, the extradural length (76.00 ± 23.28 mm) corresponds closely with that reported by Nasr et al. (74.8 ± 8.1 mm cadaveric and 75.8 ± 10 mm MRI) [8], although it is shorter than that reported by Buloz-Osorio et al. (106.64 ± 12.21 mm) (Table 5) [10].

**Table 5:** Comparison of the length and diameter of the intradural and extradural FT, VCL, and CH with other studies. FT: filum terminale, SI: stillborn infants, E: embalmed, F: fresh, MRI: magnetic resonance imaging, CH: cadaver height, VCL: vertebral column length

Authors	Sample type	Sample size	FT-L	FTI-L	FTE-L	FTI-D1	FTI-D2	FTI-D3	FTI-D4	FTI-D5	FTE-D1	FTE-D2	FTE-D3	CH	VCL
Harmeier J (1933) [30]	Unspecified	-	-	-	80	-	-	-	-	-	-	-	-	-	-
Tarlov IM (1938) [31]	Unspecified	-	-	-	75	-	-	-	-	-	-	-	-	-	-
Pinto et al. (2002) [5]	Cadavers (F)	41	-	156.44 (112.8-211.1)	-	1.38 (0.4-2.5)		0.76 (0.1-1.55)	-	-	-	-	-	-	-
Tubbs RS et al. (2005) [11]	Cadaver (E)	15	-	-	80 (70-105)	-	-	-	-	-	-	-	-	-	-
Fontes RB et al. (2006) [29]	Cadaver (F)	20	-	155.4	-	1.56 (0.31-2.30)	-	1.03 (0.14-2.20)	-	-	-	-	-	-	-
Nasr AY (2007) [14]	Cadaveric	10	247.3 ± 35.2 (196.2-293.8)	165.3 ± 26.9 (121.16-196.25)	85.7 ± 7.9 (75.56- 97.51)	1.46 ± 0.27	-	-	-	0.6070 ± 0.20	-	-	0.49 ± 0.17	168 ± 5.46 (154-182)	-
	MRI	66	230.4 ± 0.4 (90.5-220.5)	161.5 ± 0.3 (90.5-220.5)	73.6 ± 2.7 (40.5-110)	1.72 ± 0.29	-	-	-	0.96 ± 0.29	-	-	0.49 ± 0.16	-	-
Gaddam SS et al. (2012) [3]	Cadavers (SI)	13	29 (21-36)	11.8 (11-13)	-	-	-	-	-	-	-	-	-	-	-
De Vloo P et al. (2016) [12]	Cadavers (E & F)	20	-	160.01	69.86	1.91	0.92	0.8	0.68	0.68	1.07	0.73	1.02	-	-
Patel M et al. (2018) [21]	Cadaver (F)	5	-	52.2 (41-50)	77 (66-88.3)	0.38	-	-	-	-	0.60	-	-	-	-
Nasr AY et al. (2018) [8]	Cadavers (E)	25	202.9 ± 20.9	162.3 ± 12.8	74.8 ± 8.1	1.7 ± 0.2	-	0.74 ± 0.14	-	-	-	0.48 ± 0.14	-	167 ± 8.2	-
	MRI	100	248.2 ± 23.4	172.5 ± 20.6	75.8 ± 10	1.59 ± 0.26	-	0.89 ± 0.21a	-	-	-	0.45 ± 0.13	-	166.7 ± 7.3	-
Picart T et al. (2018) [1]	Cadaver (F)	10	-	167.13 ± 21.69 (135-190)	87.59 ± 17.46 (71.11-127)	1.84 ± 0.34	0.88 ± 0.20	0.71 ± 0.22	0.67 ± 0.30	0.74 ± 0.24	1.05 ± 0.38	0.77 ± 0.22	0.59 ± 0.24	-	-
Buloz-Osorio EB et al. (2025) [10]	Cadaver (E)	28	254.32 ± 26.46	152.75 ± 22.02	106.64 ± 12.21	1.66 ± 0.38	1.24 ± 0.28	0.90 ± 0.37	0.83 ± 0.34	1.12 ± 0.31	1.66 ± 0.30	1.32 ± 0.22	1.49 ± 0.51	-	-
Present study	Cadavers (E)	30	221.83 ± 24.90	148.17 ± 23.02	76.00 ± 23.28	1.67 ± 0.07	1.25 ± 0.06	0.90 ± 0.05	0.83 ± 0.05	0.98 ± 0.06	1.23 ± 0.04	1.00 ± 0.04	0.89 ± 0.05	161.93 ± 6.84	65.00 ± 4.28

The diameter measurements in the present study are consistent with those in previous research. The proximal intradural diameter (FTI-D1) measured 1.67 ± 0.07 mm, which is nearly identical to that reported by Nasr et al. (1.7 ± 0.2 mm) [8] and Buloz-Osorio et al. [10] (1.66 ± 0.38 mm) [10]. The mid-segment intradural diameter (FTI-D3:0.90 ± 0.05 mm) corresponds precisely with that reported by Buloz-Osorio et al. (0.90 ± 0.37 mm) [10] and the MRI findings by Nasr et al. (0.89 ± 0.21 mm) [8]. Notably, none of the diameters in the present study exceeded 2 mm, which is widely cited as the radiological threshold suggestive of pathological thickening in TCS [21]. This supports the reliability of the established diagnostic cutoff.

Extradural diameters (FTE-D1: 1.23 ± 0.04 mm; FTE-D2: 1.00 ± 0.04 mm; FTE-D3: 0.89 ± 0.05 mm) were within the physiological ranges described by De Vloo et al. [12] and Picart et al. [1], although Buloz-Osorio et al. [10] reported relatively larger distal extradural values, possibly reflecting differences in measurement landmarks or specimen characteristics. Earlier in 1933 and 1938, respectively, Harmeier [30] and Tarlov [31] found FTE-L was 80 mm and 75 mm, which is quite similar (Table 5). The FT plays a critical biomechanical role in maintaining the longitudinal tension balance of the SC. A thickened or fatty FT, typically defined radiologically as >2 mm in diameter, has long been considered a diagnostic indicator of tethered cord syndrome. Nevertheless, recent studies have proposed that even a radiologically normal filum may exert pathological traction if its intrinsic architecture is altered, particularly due to excessive fibrous tissue deposition and reduced elasticity [8,14,27]. Therefore, an accurate morphometric evaluation of the FT, including its total length, intradural and extradural components, and segmental diameters, is essential in distinguishing physiological variations from pathological thickening. Establishing normative anatomical values is particularly important for interpreting borderline MRI findings and guiding surgical decision-making.

Regarding the position of the FTI within the DS, the present study demonstrated 100% midline insertion. This finding aligns with that of Pokanan et al. (100%) [6] but differs from those of Hansasuta et al. (88.9% midline) [7] and Picart et al. (80% midline) [1]. Buloz-Osorio et al. [10] reported only 47.5% midline positioning, suggesting population or methodological variability (Table 6). Importantly, all measured FT diameters in this study remained below 2 mm, supporting the previously suggested physiological thresholds. The coordinated tapering pattern observed in intradural diameters, along with strong intersegmental correlations, suggests structured morphometric scaling of the FT. This finding supports the hypothesis that pathological tethering may result not only from increased thickness but also from altered structural elasticity.

**Table 6:** Comparison of midline and lateral FTI fusion in the dural sac with other studies. FTI: filum terminale internum

Authors	Sample size	FTI midline (%)	FTI left (%)	FTI right (%)
Hansasuta A et al. (1999) [7]	27	24 (88.9)	1 (3.7)	2 (7.4)
Picart T et al. (2019) [1]	10	8 (80)	1 (10)	1 (10)
Buloz-Osario EB et al. (2025) [10]	38	18 (47.5)	16 (42.8)	4 (10.7)
Pokanan S et al. (2019) [6]	83	83 (100)	-	-
Present study	30	30 (100)	-	-

A major strength and unique contribution of the present study is the simultaneous evaluation and statistical correlation of TLSC, LSCCM, VCL, FT morphometry, and CH within the same cadaveric series. Although previous studies have independently reported conus termination levels or FT dimensions, none have systematically integrated absolute SC measurements and VCL with detailed segmental FT morphometry in a single analytical framework. The present study demonstrated strong positive correlations between the VCL and TLSC, as well as between the TLSC and FT dimensions, indicating proportional anatomical scaling. Additionally, CH was significantly associated with SC parameters, further supporting the biomechanical interdependence between these structures. This comprehensive morphometric correlation analysis provides novel anatomical insights into the SC-FT-vertebral proportional relationships, which have not been previously emphasized in cadaveric or MRI-based literature. The inclusion of VCL as a measurable parameter represents a distinctive methodological advancement and constitutes the principal highlight of this study.

Limitations

This study is limited by its embalmed cadaveric design and potential selection bias toward older individuals. Embalming may influence tissue elasticity and morphometric accuracy. The relatively modest sample size may limit the detection of subtle population-based variations.

## Conclusions

The CM was most frequently terminated at the lower third of L1, and fusion of the FT to the DS predominantly occurred at the upper third of S2. All FT diameters remained below the 2 mm pathological threshold criteria for tethered cord syndrome. This study uniquely integrates TLSC, LSCCM, VCL, CH, and detailed segmental filum morphometry within a single analytical framework. Significant positive correlations between VCL and TLSC, as well as between FT-L and proximal intradural diameter, demonstrate proportional anatomical scaling. The inclusion of vertebral column measurements and their correlation with FT dimensions represents a distinctive methodological advancement and provides clinically valuable normative data for the anatomical and surgical evaluation of tethered cord syndrome.
